# Development of a genetic results return protocol for an established Alzheimer's study: experience with the National Institute on Aging Alzheimer's Family‐Based Study

**DOI:** 10.1002/alz70858_101090

**Published:** 2025-12-25

**Authors:** Amanda Chan, Malia C. Rumbaugh, Dolly Reyes‐Dumeyer, Pamela Del Rosario, Tatiana M. Foroud, Richard Mayeux

**Affiliations:** ^1^ Taub Institute for Research on Alzheimer's Disease and the Aging Brain, Columbia University, New York, NY, USA; ^2^ Department of Neurology, Vagelos College of Physicians and Surgeons, Columbia University, New York, NY, USA; ^3^ G.H. Sergievsky Center, Columbia University, New York, NY, USA; ^4^ Department of Medical and Molecular Genetics, Indiana University School of Medicine, Indianapolis, IN, USA; ^5^ Columbia University Irving Medical Center, New York, NY, USA

## Abstract

**Background:**

As sequencing technologies allow for faster and easier access to genomic data, the incorporation of genetic information into research studies has become more routine. In conjunction, there is a push to return relevant genetic results to research participants. However, there are many considerations for genetic results return that may not apply to other medical information including CLIA‐certification, genetic counseling, and familial implications. This project aims to create a scalable protocol for genetic results return that can be implemented into an existing project.

**Method:**

The Alzheimer's disease Family‐Based study (AD‐FBS) started in 2003 and has recruited over 1,400 families. While research genetic sequencing was always a study component, participants did not historically receive these results. In the study's newest phase, six genes associated with autosomal dominant dementia (*APP*, *PSEN1*, *PSEN2*, *MAPT*, *GRN*, *C9orf72*) are returned to participants with early‐onset dementia.

**Result:**

The protocol consists of four components: (1) recruitment and consent (2) pre‐test genetic counseling (3) genetic testing (4) result disclosure and post‐test counseling. Eligibility criteria is a clinical diagnosis of AD or MCI and age of onset 65 or under. Since the FBS study recruits multiplex families, one individual (person with the earliest onset) per family is eligible for results disclosure. Informed consent is a key part of the protocol and the participant (or health care proxy) can decline the return of results at any point before results disclosure. Participants are provided with pre‐ and post‐test genetic counseling to thoroughly understand the context of testing and implications of results for themselves and their family members. Genetic testing is performed at a CLIA‐certified lab and positive, negative, and variants of uncertain significance are returned. Participants are given surveys before and after results disclosure to assess the psychosocial impact of the genetic testing process. Materials developed include a participant‐facing handout on dementia genetic testing, genetic result return consent form, pre‐ and post‐ questionnaires, and genetic results letters. Preliminary responses indicate participants are generally satisfied with the genetic testing process, regardless of the result received.

**Conclusion:**

Our project demonstrates the feasibility of incorporating genetic results return in a pre‐existing research study.

